# Study on the Spatiotemporal Changes and Driving Factors of Habitat Quality in the Yarlung Zangbo River From 2000 to 2020

**DOI:** 10.1002/ece3.70807

**Published:** 2025-01-31

**Authors:** Yu Chen, Yujie Kang, Jingji Li, Yanguo Liu, Qin Liu, Zhengyu Luo, Xiaohui Zhou, Tingbin Zhang, Guoyan Wang, Xiaolu Tang, Xiangjun Pei

**Affiliations:** ^1^ State Key Laboratory of Geological Hazard Prevention and Geological Environmental Protection Chengdu University of Technology Chengdu China; ^2^ College of Geography and Planning Chengdu University of Technology Chengdu China; ^3^ College of Ecology and Environment Chengdu University of Technology Chengdu China; ^4^ School of Environment Resource Southwest University of Science and Technology Mianyang China; ^5^ Institute of Mountain Hazards and Environment, Chinese Academy of Sciences Chengdu China; ^6^ College of Earth Science Chengdu University of Technology Chengdu China

**Keywords:** driving factors, habitat quality, land use, optimal parameter geographic detector (OPGD), partial least squares structural equation model, Yarlung Zangbo River

## Abstract

The Yarlung Zangbo River (YLZB), the world's highest plateau river, possesses a particularly fragile ecosystem, making it highly vulnerable to global climate change. Understanding changes in YLZB habitat quality and their driving mechanisms is crucial for ecological protection and sustainable development in the region. Based on land use data from 2000 to 2020, we conducted a quantitative study on the spatiotemporal changes and driving mechanisms of habitat quality in the YLZB. This study employed habitat quality model, Land Use Transition Matrix, optimal parameter geographical detector, and partial least squares structural equation model (PLS‐SEM). The results show that: (1) Forests, grasslands, and unused land account for 94.14% of the basin area. The areas of unused land, forest land, and water bodies have continuously increased, while the areas of grasslands, permanent glaciers, and snowfields have continuously decreased. The decline was most pronounced from 2005 to 2010. (2) The habitat quality in the study area is higher in the southeast and lower in the west. The area of degraded habitats is significantly larger than that of improved habitats. (3) NDVI, elevation, and annual average temperature are key factors affecting changes in habitat quality. Elevation indirectly affects NDVI by influencing climate conditions, leading to a decline in habitat quality. This study provides a scientific basis for understanding ecological trends in YLZB habitat quality, it provides new insights into the intrinsic driving mechanisms in high‐altitude regions, and it offers theoretical support for relevant departments to implement sustainable management and conservation efforts.

## Graphical abstract

This study uses land use data from 2000 to 2020 and employs ArcGIS 10.8 and InVEST to construct the spatiotemporal patterns of habitat quality in the YLZB. Furthermore, methods such as optimal parameter geographic detectors, partial least squares structural equation model (PLS‐SEM), and correlation analysis are used to quantify the direct and indirect effects of key driving factors on habitat quality changes.

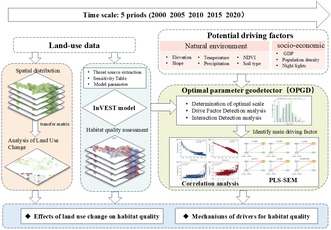

## 1 Introduction

Habitat quality refers to the ability to provide the natural resources necessary for individual survival and sustainable population development within a certain spatial and temporal scope. The level of habitat quality is a key indicator for assessing ecosystem health and habitat suitability (Song et al. 2021; Zheng et al. 2022). Under the combined effects of global climate change and human activities, ecosystem degradation has become a significant challenge to maintaining ecological security in the Earth's environmental system and achieving sustainable human development (Fu 2022). Global warming has led to a rapid temperature increase in the Qinghai‐Tibet Plateau, triggering imbalances such as accelerated glacier retreat, significant lake expansion, and increased glacial runoff (Chen et al. 2021). Under the impetus of policies like “New Urbanization Planning” and “Western Development,” activities such as rapid urbanization, deforestation, and farmland expansion have significantly increased human disturbances (Zhang, Liu, et al. 2019). The resource occupation, pollution, habitat fragmentation, and land use changes caused by these climate changes and human activities are placing increasing pressure on natural systems, thereby affecting the stability of watershed habitat quality in high‐altitude regions (Zheng, Wang, and Li 2023; Deng 2023; Chen et al. 2021). Changes in land use, the most significant driver of habitat quality variations, illustrate how ecosystems temporally and spatially adapt to environmental alterations under the influence of natural factors and human activities (Aguilar et al. 2019; Fu et al. 2005). Consequently, investigating watershed‐scale habitat quality dynamics and their underlying causes facilitates a clearer comprehension of ecosystem degradation patterns and their mechanisms of response to environmental changes.

In recent years, habitat quality assessment has emerged as a focal point in ecological security studies worldwide (Bai et al. 2019; Mengist, Soromessa, and Feyisa 2021; Xiang et al. 2023). Field surveys and model simulations are the primary methods for assessing habitat quality (Yu et al. 2020). However, field surveys are challenging due to their dependence on long‐term species monitoring data, limiting their suitability for large‐scale and long‐term studies. As a result, researchers have developed various habitat assessment models tailored to these research needs. These models, characterized by their modeling, spatial analysis, and quantification capabilities, are ideal for studying long‐term spatial and temporal patterns of habitat quality at watershed or larger scales. Prominent among these models are the Habitat Suitability Index (HSI), Social Values for Ecosystem Services (SolVES), and Integrated Valuation of Ecosystem Services and Trade‐offs (InVEST) models, which are extensively used for large‐scale habitat quality assessments (Zhang et al. 2019; Li, Zhou, et al. 2021; Sharp et al. 2018). The InVEST model, known for its accessibility to data, precise analytical tools, quantifiable results, and spatial visualization capabilities, stands out as a comprehensive tool widely applied in watershed and regional habitat assessments (Shang et al. 2021). Researchers globally have explored habitat quality from various angles, including regional disparities (Ahmadi Mirghaed and Souri 2021), the impact of land use (Berta Aneseyee et al. 2020), biodiversity conservation based on habitat assessments (Guo, Zhang, and Li 2010), and the mechanisms driving habitat quality changes (Dai et al. 2019). Previous studies have provided a solid foundation for understanding habitat quality variations under different land uses, terrains, and vegetation types. However, existing research primarily focuses on global or regional scales, with insufficient attention given to specific areas, especially the impacts of climate change and human activities on habitat quality in high‐altitude watersheds. This highlights the lack of studies on the habitat quality of the Yarlung Zangbo River. To address this gap, this study aims to identify trends in habitat quality changes in the Yarlung Zangbo River watershed from the perspective of climate change and human activities and further explore the mechanisms driving these changes, which hold significant scientific and practical relevance.

The Yarlung Zangbo River (YLZB) represents a globally significant high‐altitude ecosystem, with its unique existence closely linked to the ecological security of the Qinghai‐Tibet Plateau and its surrounding regions (Li, Yang, and Zhang 2023; Fan and Fang 2022). In recent years, the long‐term impacts of global climate change and human activities have intensified the ecological challenges of the Qinghai‐Tibet Plateau, particularly threatening the habitat quality of the YLZB (Pritchard 2019; Xu, Gao, et al. 2020; Xu, Hu, et al. 2019; Wang et al. 2021; Liu, Xu, and Peng 2019). Based on this, the main objectives of this study are: (1) to assess the spatiotemporal characteristics of habitat quality changes in the YLZB; (2) to evaluate the spatiotemporal relationships between land use changes and habitat quality; and (3) to reveal the driving mechanisms of key factors influencing habitat quality. The results are expected to provide decision‐making support for ecological construction and economic development in the YLZB region and offer a scientific basis for subsequent ecological restoration planning and implementation.

## 2 Materials and Methods

### 2.1 Methodology

**FIGURE 1:**
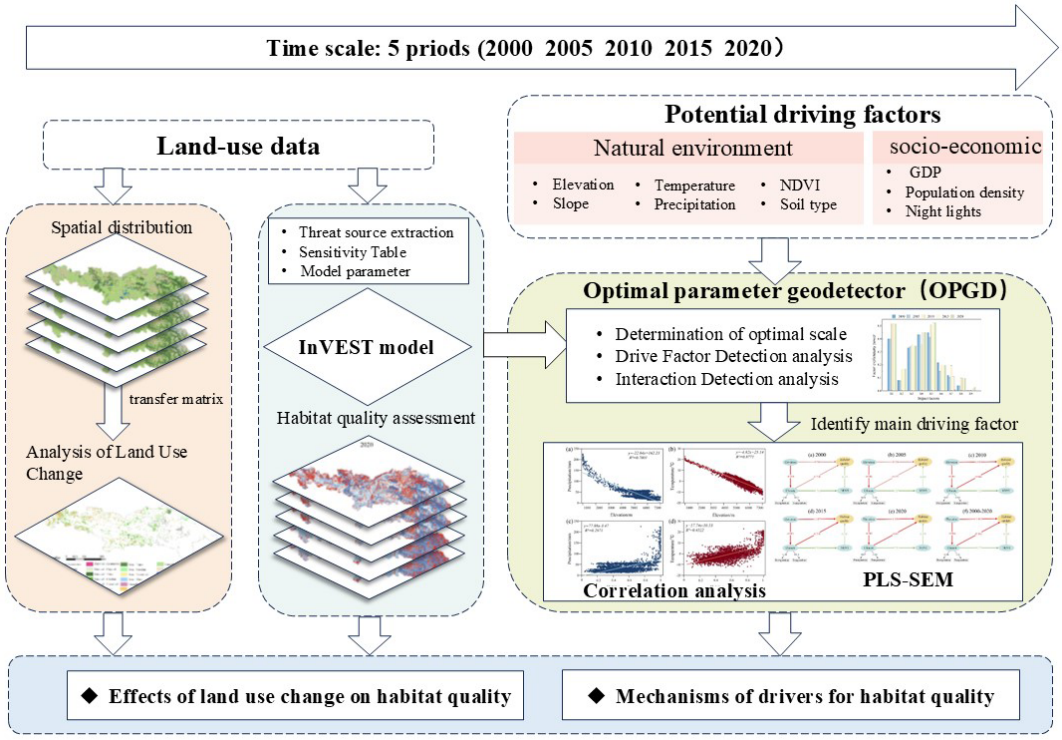
Schematic of methodological framework.

The theoretical framework shown in Figure 1 serves as the foundation for studying changes in habitat quality and the driving mechanisms in the YLZB. Based on landscape ecology theory and relevant studies (Yu et al. 2024; Jia et al. 2024), this study first applies the InVEST model to quantitatively assess habitat quality in the YLZB. Then, a land transfer matrix is employed to examine the spatial distribution of land use from 2000 to 2020. Subsequently, the GeoDetector model is used to identify key factors influencing habitat quality. Finally, the PLS‐SEM model is applied to further explore the driving mechanisms between key factors and habitat quality.

### 2.2 Study Area

**FIGURE 2:**
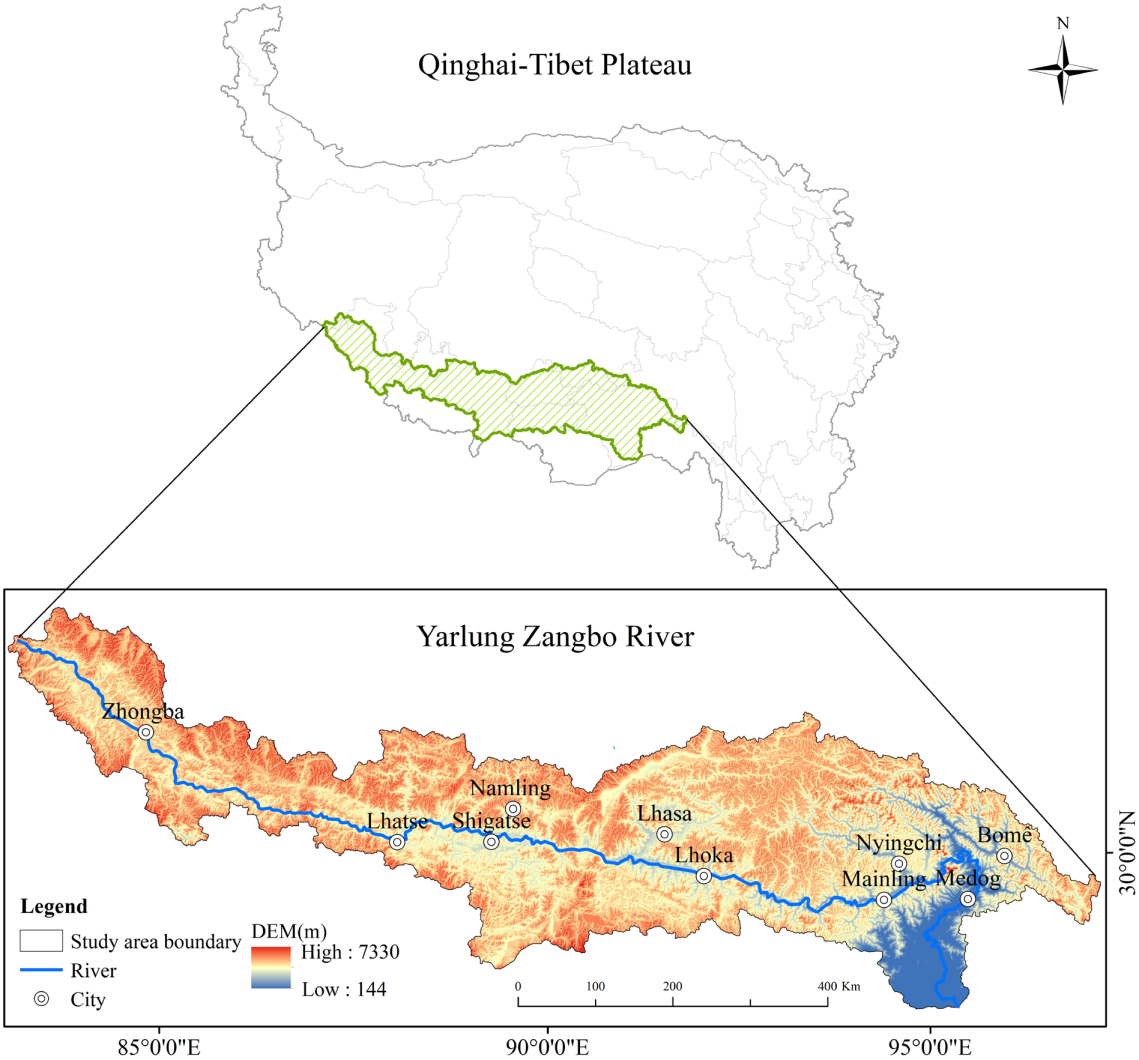
Location, range, and elevation of the study area.

The Yarlung Zangbo River is situated in the southern Qinghai‐Tibet Plateau (Figure 2), flanked by the Gangdise‐Nyenchen Tanglha Mountains and the Himalayas. It spans geographically from approximately 27°80′ N to 31°16′ N and 82°00′ E to 97°07′ E, encompassing diverse climatic zones including arid, humid, and semi‐arid to semi‐humid areas, with an average elevation exceeding 4000 m (Xu, Ban, and Zhang 2022; Huang et al. 2019). Originating from the Jiemayangzong Glacier in the northern foothills of the Himalayas, the YLZB is one of the world's highest‐altitude rivers, stretching over 2840 km with abundant watershed flow. It boasts an annual average runoff of 1651 m^3^, ranking second only to the YLZB in hydroelectric potential and being Tibet's largest river (Liu et al. 2023). Spanning widely from east to west, the basin's terrain slopes steeply from high in the west to low in the east, resulting in notable climatic variations. Rainfall distribution is uneven, with the rainy season concentrated mainly from June to September, contributing about 60% of the annual precipitation. The upper reaches, from Weijiema Yangzong Qu to Lizhi, feature expansive plateau valleys dominated by meadows and grasslands, receiving less than 300 mm of annual average precipitation and ample sunshine. The middle reaches, extending from Lizhi to Milin County, comprise river valleys with prevalent meadows and alpine vegetation, receiving annual average precipitation ranging from 300 to 600 mm and maintaining an average annual temperature of 5.2°C. In the lower reaches, from Milin County to the exit in the canyon area, shrubs and mixed needle‐leaf and broadleaf forests dominate, with annual average precipitation around 2000 mm and a basin‐wide average temperature varying from −7.1°C to 22.2°C, increasing downstream (Chen et al. 2016; Yu and Zhang 2020). The upper, middle, and lower reaches respectively account for 13%, 68%, and 24% of the total river length.

### 2.3 Data Sources and Processing

Data sources include land use data for five periods (2000, 2005, 2010, 2015, and 2020) in the study area, a digital elevation model (DEM), slope, natural environmental factors (mean annual precipitation, mean annual temperature, normalized vegetation index (NDVI), and soil types), and socioeconomic data (population density, GDP, and nighttime lights) (Table 1). Based on land use classification standards, the watershed land use types are divided into seven primary categories (cropland, forestland, grassland, water bodies, built‐up land, unused land, and permanent glacier and snow cover) and 24 secondary categories (Table 3). Slope data were extracted from DEM using the slope tool in ArcGIS 10.8. Combining previous research and practical conditions (Li, Zou, et al. 2021), a 9000 × 9000 grid was created using the fishnet tool in ArcGIS 10.8. The zonal statistics tool was used to obtain the mean values of various driving factors, serving as the smallest analysis unit and data carrier. To ensure spatial precision consistency across all data, the projection coordinate system was standardized to WGS_1984, with a spatial resolution of 30 m.

**TABLE 1:** Datasets used in the study and their sources.

Name of data		Accuracy	Source
Land use data	Land use types (CLCD)	30 m	Chinese Academy of Sciences Resource and Environmental Science Data Center (https://www.resdc.cn/)
Natural environment data	Digital Elevation Model (DEM)	30 m	Geospatial Data Cloud (http:/www.gscloud.cn/)
	Annual average precipitation (Pre)	1 km	National Tibetan Plateau Data Center (https://data.tpdc.ac.cn/zh‐hans/)
	Annual average temperature (Tem)	1 km	National Tibetan Plateau Data Center (https://data.tpdc.ac.cn/zh‐hans/)
	Normalized Vegetation Index (NDVI)	500 m	EARTHDATA (https://www.earthdata.nasa.gov/)
	Soil types (Soil)	1 km	Chinese Academy of Sciences Resource and Environmental Science Data Center (https://www.resdc.cn/)
Socioeconomic data	Gross Domestic Product (GDP)	1 km	Data Center of the Institute of Geographic Sciences and Natural Resources Research, Chinese Academy of Sciences (https://www.resdc.cn/)
	Population density (Pop)	1 km	LANDSCANS (https://landscan.ornl.gov/)
	Nighttime lights (NTL)	500 m	National Earth System Science Data Center (http://www.geodata.cn/)

#### 2.3.1 Habitat Quality Assessment

The study employs the Habitat Quality Model within the InVEST framework, which integrates the sensitivities of different land use types and external threat factors to compute habitat quality (Nematollahi et al. 2020). This model calculates the Habitat Quality Index to evaluate the spatial distribution of habitat quality across various periods. The calculation formula (1) is:

(1) $$Q_{\mathrm{xj}}=H_{j}\left(1-\left(\frac{D_{\mathrm{xj}}^{z}}{D_{\mathrm{xj}}^{z}+k^{z}}\right)\right)$$

*Q*_*xj*_ is the habitat quality index for grid cells of habitat type *j*, ranging from 0 to 1 where higher values indicate higher habitat quality; *H*_*j*_ is habitat suitability; *z* is a normalization constant, typically set to 2.5; *k* is the half‐saturation parameter, typically half of the maximum value of *D*_*xj*_; *D*_*xj*_ is habitat degradation, indicating the degree of habitat degradation. The calculation formula (2) is:

(2) $$D_{\mathrm{xj}}=\sum_{r=1}^{R}\sum_{y=1}^{Y_{r}}\left(\frac{W_{r}}{\sum_{r=1}^{R}W_{r}}\right)r_{y}i_{\mathrm{rxy}}\beta_{x}S_{\mathrm{jr}}$$

*r* is the stress factor of the habitat; *y* is the stress factor grid; *W*_*r*_ is the weight of stress factor *r*; *i*_*rxy*_ is the stress level of *r*_*y*_ on habitat grid *x*; *β*_*x*_ is the accessibility level of grid *x*; *S*_*jr*_ is the sensitivity of habitat type *j* to stress factor *r*, ranging from 0 to 1 where higher values indicate greater sensitivity to habitat degradation. The calculation formulas (3) and (4) are as follows:

(3) $$i_{\mathrm{rxy}}=1-\left(\frac{d_{\mathrm{xy}}}{d_{r\max}}\right)\;\text{if linear}$$

(4) $$i_{\mathrm{rxy}}=\exp\left(-\left(\frac{2.99}{d_{r\max}}\right)d_{\mathrm{xy}}\right)\;\text{if}\;\text{exponential}$$

*d*_*xy*_ is the straight‐line distance between grid *x* and grid *y*; *d*_*r*max_ is the maximum influence distance of stress factor *r*.

Threat sources refer to types that have a more significant and destructive impact on the habitat quality of the area. Based on the InVEST model manual (Sharp et al. 2018) and related literature (Hou et al. 2021; Huang et al. 2023), cropland, urban land, rural settlements, other construction land, and bare land with low habitat quality, which are significantly affected by human activities, were selected as threat sources (Tables 2 and 3).

**TABLE 2:** Weight assignment and maximum impact distance of threat factors.

Threat	Max_dist/km	Weight	Decay
Cropland	3	0.2	Linear
Townland	8	1	Exponential
Rural settlements	5	0.6	Exponential
Other construction land	5	0.5	Linear
Unused land	1	0.3	Linear

**TABLE 3:** Sensitivity of different land types to threat factors.

Land use type	Habitat suitability	Cropland	Townland	Rural settlements	Other construction land	Unused land
Paddy field	0.4	0.3	0.35	0.3	0.3	0.2
Arid	0.3	0.3	0.4	0.5	0.5	0.2
Woodland	1	0.8	0.8	0.8	0.8	0.3
Low wood	1	0.4	0.45	0.4	0.6	0.3
Open woodland	1	0.85	0.9	0.85	0.5	0.3
Other woodlands	0.8	0.9	0.95	0.9	0.7	0.3
High‐cover grassland	0.7	0.4	0.45	0.4	0.5	0.4
Medium‐cover grassland	0.6	0.45	0.5	0.45	0.5	0.5
Low‐cover grassland	0.5	0.5	0.55	0.5	0.5	0.6
Waterway	1	0.65	0.7	0.65	0.9	0.4
Lakes	0.9	0.7	0.75	0.7	0.9	0.4
Reservoir pit	0.9	0.7	0.75	0.7	0.9	0.4
Permanent glacial snow	1	0.6	0.8	0.6	0.8	0.2
Mudflat	0.6	0.75	0.8	0.75	0.8	0.2
Townland	0	0	0	0	0	0
Rural settlements	0	0	0	0	0	0
Other construction land	0	0	0	0	0	0
Sandy land	0.1	0.1	0.3	0.4	0.5	0
Gobi	0.1	0.1	0.4	0.2	0.4	0
Saline soil	0.1	0.2	0.2	0.2	0.2	0
Marshland	0.6	0.7	0.75	0.7	0.75	0
Bare ground	0	0	0	0	0	0
Bare rock texture	0.1	0.1	0.3	0.1	0.3	0.1
Other	0.7	0.05	0.05	0.05	0.05	0.1

#### 2.3.2 Optimal Parameters Geodetector (OPGD)

GeoDetector is a spatial statistical model used to detect spatial heterogeneity and identify driving forces (Wang and Xu 2017). Using the “OPGD” package in R (Song et al. 2020), multiple discretization methods were applied to different continuous variables to select the optimal spatial discretization method for each variable. This study mainly utilizes single‐factor detection and interaction detection. Single‐factor detection identifies the explanatory power of individual factors on habitat quality, while interaction detection examines whether the combined effect of two factors amplifies or diminishes their influence on habitat quality (Table 4). The calculation formula (5) is as follows:

(5) $$q=1-\frac{\sum_{h=1}^{L}N_{h}\sigma_{h}^{2}}{{\mathrm{N\sigma}}^{2}}$$

*q* represents the explanatory power of each factor on habitat quality, ranging from 0 to 1; a higher *q* value indicates stronger explanatory power, whereas a lower value indicates weaker power. *L* denotes the stratum of variable *Y* or factor *X*. *N*_*h*_ and *N* represent the number of units corresponding to habitat quality and factors in the *h*‐th stratum and the entire region, respectively. $\sigma_{h}^{2}$ and *σ* ^2^ represent the variance of habitat quality change in the *h*‐th stratum and the entire region.

**TABLE 4:** Types of interaction of the detection factors.

Basis of judgment	Interaction
$q\left(X_{1}\cap X_{2}\right)<\mathrm{Min}\left[q\left(X_{1}\right),q\left(X_{2}\right)\right]$	Nonlinear attenuation
$\mathrm{Min}\left[q\left(X_{1}\right),q\left(X_{2}\right)\right]<q\left(X_{1}\cap X_{2}\right)<\mathrm{Max}\left[q\left(X_{1}\right),q\left(X_{2}\right)\right]$	Single‐factor nonlinear attenuation
$q\left(X_{1}\cap X_{2}\right)>\mathrm{Max}\left[q\left(X_{1}\right),q\left(X_{2}\right)\right]$	Bi‐factor enhancement
$q\left(X_{1}\cap X_{2}\right)>q\left(X_{1}\right)+q\left(X_{2}\right)$	Nonlinear enhancement
$q\left(X_{1}\cap X_{2}\right)=q\left(X_{1}\right)+q\left(X_{2}\right)$	Stand alone

*Note: X*_1_ and *X*_2_ indicate influencing factors of habitat quality functions.

Considering the unique natural conditions and socioeconomic attributes of YLZB, with habitat quality as the dependent variable, we selected 9 driving factors as independent variables. These factors include natural environmental and socioeconomic conditions: elevation, slope, mean annual precipitation, mean annual temperature, NDVI, soil type, GDP, population density, and nighttime light data. This study involves analyzing the contribution rates of these factors and detecting their interactions. The GeoDetector model, as an excellent analytical tool in this regard, can reveal the main driving factors of spatial differentiation in YLZB and their interactions.

#### 2.3.3 Partial Least Squares Structural Equation Model (PLS‐SEM)

PLS‐SEM, as a multivariate statistical analysis technique, evaluates the influence of observed variables on latent variables through a pre‐specified structural model and quantifies the interactions between latent variables, thereby providing a deeper analysis of the complex relationships between variables (Wei et al. 2019; Richter et al. 2015). The PLS‐SEM model can effectively reveal the complex relationships between habitat quality and multiple factors such as natural elements and human activities, and it can identify and quantify the impact intensity and pathways of these factors on habitat quality (Dai et al. 2024). Although covariance‐based techniques (CB‐SEM) and partial least squares PLS‐SEM are both structural equation models (SEM) based on the same principles. However, compared to CB‐SEM, the advantage of PLS‐SEM is that it relaxes the requirement for normally distributed data and is not limited by sample size (Hair et al. 2014; Shi, Lee, and Maydeu‐Olivares 2019). In this study, the key driving factors were identified through factor detection based on the GeoDetector, and a PLS‐SEM model was established using SmartPLS 4.0 software to analyze the direct and indirect effects of the key driving factors on habitat quality, providing insights into the underlying mechanisms driving habitat quality changes. The external estimation of latent variables is used to perform the internal estimation of latent variables *γ*_*i*_ and *γ*_*j*_ associated with each other.

(6) $$\gamma_{j}=\sum_{i=1}^{i}\;\beta_{\mathrm{ji}}\gamma_{i}+\delta_{j}$$

The external estimation of latent variable *γ*_*j*_ is established as a linear combination of manifest variables *x*_*jk*_:

(7) $$x_{\mathrm{jk}}=\varphi_{\mathrm{jk}}\gamma_{j}+\varepsilon_{\mathrm{jk}}$$

where *γ*_*j*_ is the vector volume coefficient *φ*_*jk*_. Variable *x*_*jk*_ is standardized.

## 3 Results

### 3.1 Spatial and Temporal Patterns of Land Use Change

**FIGURE 3:**
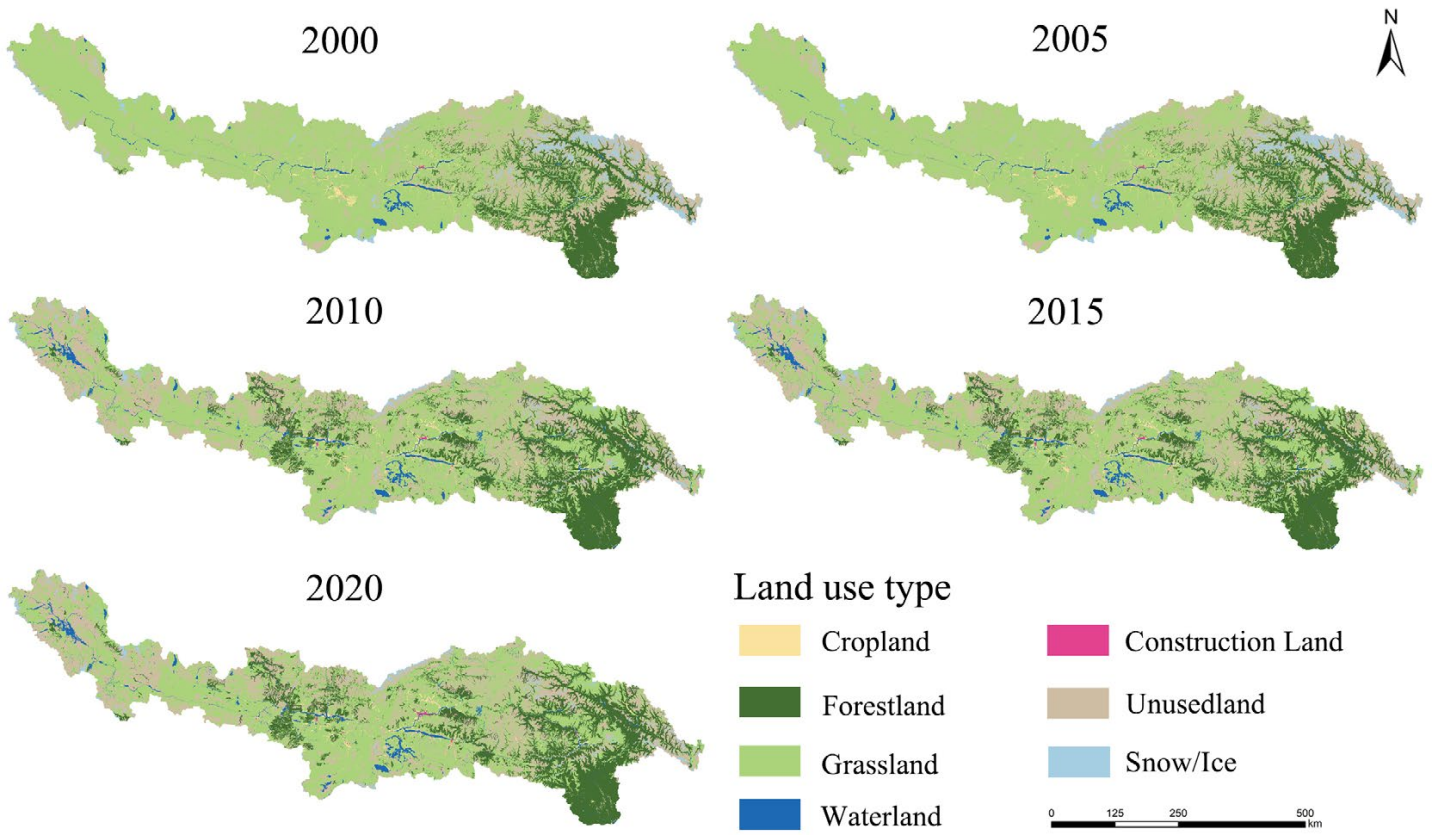
Spatial distribution of land use from 2000 to 2020.

**FIGURE 4:**
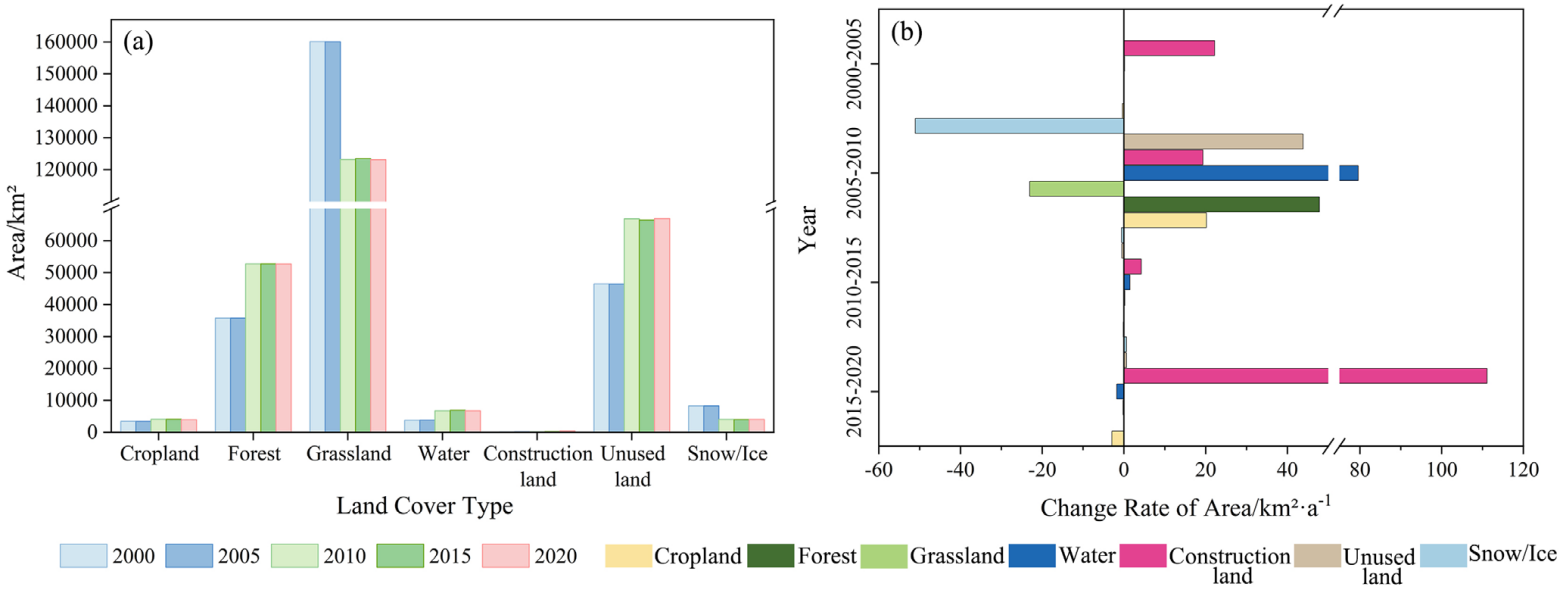
(a) Changes in land use types from 2000 to 2020 and (b) rates of change in land use area from 2000 to 2020.

From 2000 to 2020, the spatial distribution of land use in the YLZB changed significantly. The results indicate that the study area is predominantly composed of forest land, grassland, and unused land, which together account for 94.14% of the total area (Figure 3). Forest land is primarily distributed in the downstream Linzhi area, grassland is widely spread across the upper and middle reaches' broad valleys, while permanent glaciers and snowfields, and unused land are found in high‐altitude regions. Cropland and construction lands are mostly located near water bodies and are concentrated in the middle reaches, such as Shigatse and Lhasa (Figure 1). From 2000 to 2020, the area change characteristics of different land use types in the study area varied (Figures 3 and 4). Among them, unused land (+20,429.31 km^2^), forest land (+17,027.46 km^2^), and water bodies (+2981.52 km^2^) had the largest increases and showed a significant growth trend, while arable land (+545.26 km^2^) and construction land (+261.05 km^2^) saw slight increases. Conversely, the area of grassland (−37,016.19 km^2^) and permanent glaciers and snowfields (−4212.65 km^2^) decreased significantly. As shown in Figures 3 and 4b, the most significant land use changes occurred between 2005 and 2010, with forest land increasing by 17,077.31 km^2^, at a rate of 47.8 km^2^/a, mainly in the areas surrounding Duoxiongzangbu and Lazi County. Unused land increased by 20,374.63 km^2^, with a change rate of 43.8 km^2^/a, and the expanded area was widely distributed across the upper and middle reaches. Grassland decreased by 36,937.65 km^2^, at a rate of −23.1 km^2^/a, primarily in the upper and middle reaches, including areas such as Zhongba and Shigatse. Thus, the degradation of grassland and the expansion of unused land in the western and high‐altitude regions of YLZB, as well as the accelerated glacier retreat and expansion of forest land in the eastern regions, are the outcomes of the combined impacts of human activities (such as grazing and the conversion of cropland to forest) and climate change (such as glacier retreat and increased water resources caused by global warming).

From 2000 to 2020, the study area experienced significant land use changes totaling 52,000 km^2^, primarily affecting forest land, grassland, and unused land (Table 5). Specifically, unused land, forest land, water bodies, cropland, and construction areas increased by 20,418.80, 9763.02, 2981.41, 545.03, and 261.03 km^2^ net, respectively. In contrast, grassland and permanent ice and snow fields decreased by 37,016.65 and 4216.21 km^2^ net. The expansion of forest land resulted largely from conversions from grassland, while the growth in water bodies and unused land stemmed primarily from transformations of grassland and permanent ice and snowfields. Conversely, the reduction in grassland was primarily due to conversions to forest and unused land, and the decline in permanent ice and snowfields was predominantly due to conversions to unused land. Since 2010, the pace of land use change has stabilized, signaling improved opportunities for ecological restoration. Since 2010, changes in land use types have remained relatively stable, suggesting that the environmental protection policies in YLZB have been effective in the past decade.

**TABLE 5:** Land use type transfer matrix from 2000 to 2020.

2000/2020	Cropland	Forest land	Grassland	Water	Construction land	Unused land	Snow/Ice	Total
Cropland	1451.54	523.34	979.45	214.27	117.15	126.89	—	8245.68
Forest land	370.17	26,789.6	7023.69	249.52	17.97	1235.65	14.11	160,124.20
Grassland	1451.54	19,650.27	97,184.5	2942.07	147.7	37,816.04	511.26	3412.65
Water	106.44	152.75	297.6	2949.91	15.85	238.57	0.53	118.17
Construction land	29.97	4	12.08	3.4	65.2	3.51	—	35,700.71
Unused land	127.19	5216.37	15,604.32	357.26	15.33	23,577.09	1573.27	3761.66
Snow/Ice	—	390.95	2005.92	26.64	—	3891.88	1930.3	46,470.83
Total	4029.48	123,107.6	3957.68	379.2	52,727.28	6743.07	66,889.63	257,833.9

### 3.2 Habitat Quality Index Variation Characteristics

**FIGURE 5:**
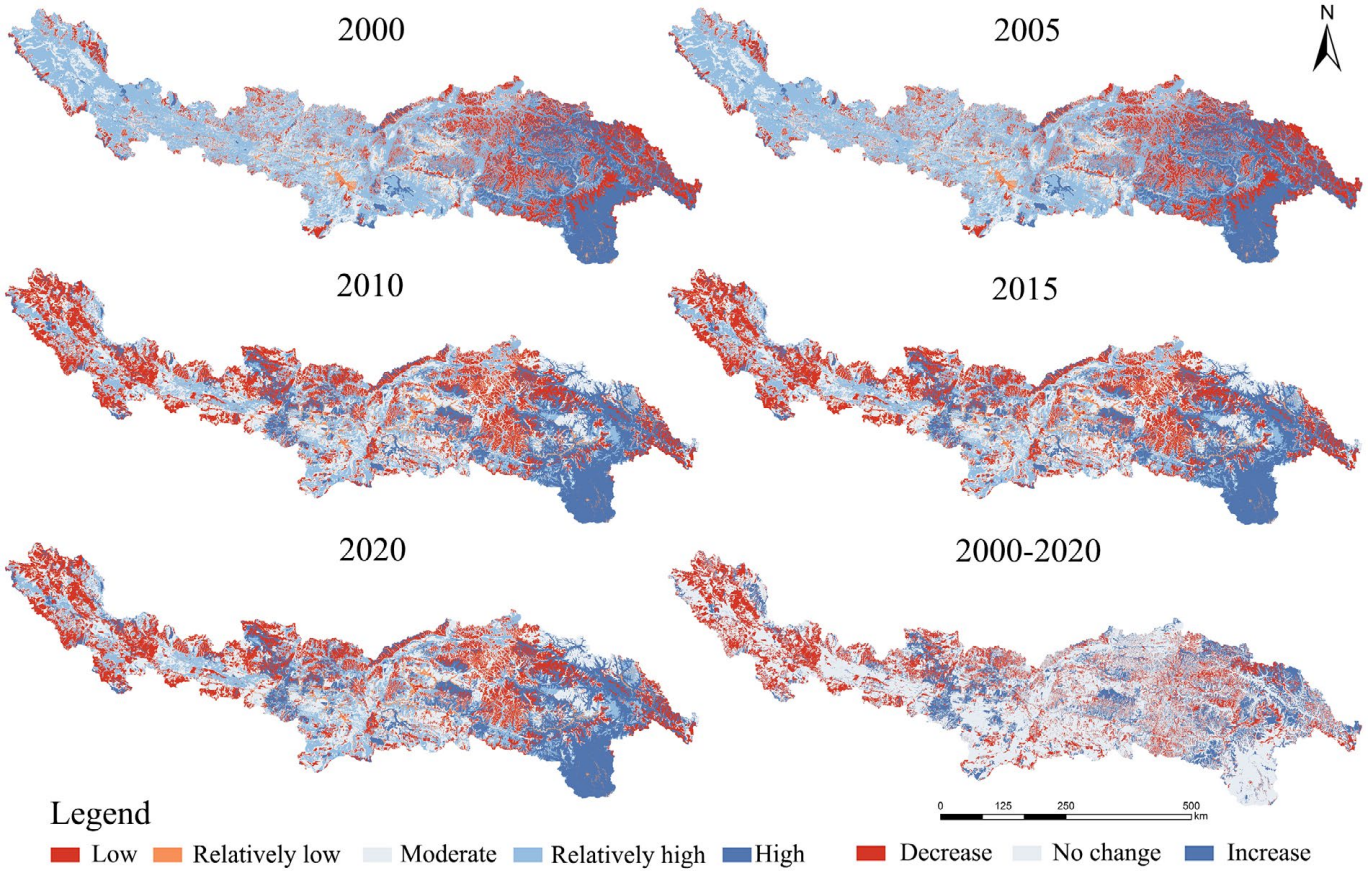
Spatial distribution of changes in habitat quality from 2000 to 2020.

**FIGURE 6:**
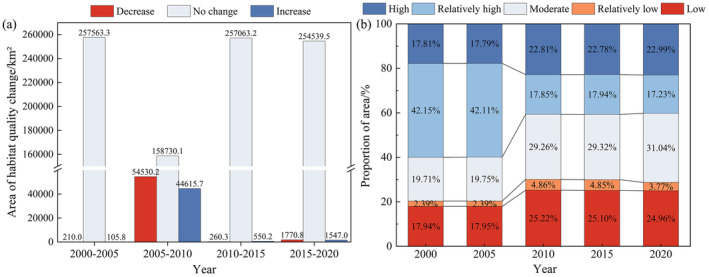
(a) Features of area increase and decrease, and (b) Changes in area of different habitat quality levels in the study area from 2000 to 2020.

From 2000 to 2020, the average habitat quality in the YLZB region showed a slight overall decline, decreasing from 0.59 in 2000 to 0.54 in 2020. The habitat quality in the study area exhibits a spatial distribution pattern of “higher in the southeast and lower in the west.” Habitat quality values are higher in low‐altitude areas such as southern Linzhi and lower in high‐altitude regions like southern Ali (Figure 5). Using the natural breaks method, habitat quality indices are classified into five levels: low (0–0.2), relatively low (0.2–0.4), moderate (0.4–0.6), relatively high (0.6–0.8), and high (0.8–1.0). Analysis of changes in area for each quality level reveals that the most significant decline occurred between 2005 and 2010, with the proportion of relatively high‐value areas decreasing by 24.26%. In contrast, areas with relatively low, low, moderate, and high values all experienced varying degrees of increase, with proportions rising by 7.27%, 2.49%, 9.51%, and 5.02%, respectively. After 2010, habitat quality improved significantly, with an increasing proportion of high and moderate‐quality areas. The proportion of relatively high‐value areas decreased noticeably, while the proportion of low and relatively low‐value areas increased by 8.4% overall, as illustrated in Figure 6.

To clarify the dynamics of habitat quality changes, we categorized these shifts into three groups: increase, stability, and decrease. A comparison of the spatial distribution characteristics of habitat quality changes in different periods (Figures 5 and 6) revealed that between 2005 and 2010, about 17.30% of the region showed an improvement in habitat quality, with the improved areas mainly concentrated in the downstream Palongzangbu River Basin, where ecological protection projects, such as natural forest conservation efforts, played a positive role (Zheng et al. 2024). Meanwhile, 21.15% of the region showed a trend of habitat degradation, with the degraded areas widely distributed in the upper and middle reaches of the study area, such as Zhongba County and Shigatse City. This may be due to the harsh irrigation conditions and climate variability in the high‐altitude regions, limited economic activities, leading to the migration of rural labor populations, abandonment of farmland, and land exposure (Zhang et al. 2023).

### 3.3 Land Use and Habitat Quality Change Characteristics

**FIGURE 7:**
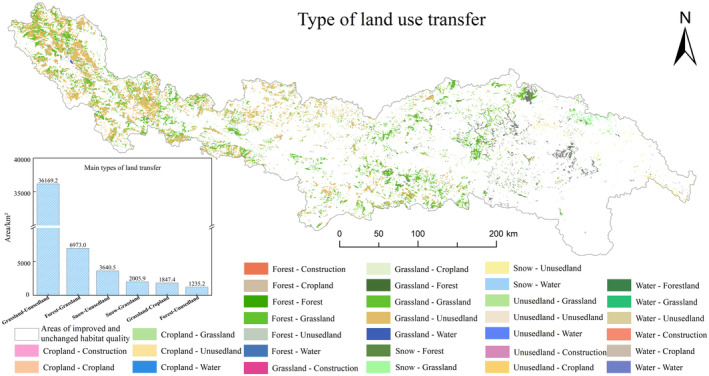
Land Use Change in Habitat Quality Degradation Areas from 2000 to 2020.

Understanding that land use changes have profound effects on regional habitat quality and its spatial distribution (Yang et al. 2018; Zheng, Xie, and You 2022; Zheng et al. 2022), to further observe the land use changes in areas of habitat quality degradation, the habitat quality degradation zones from 2000 to 2020 were overlaid with the land transfer matrix (Figure 7). The results show that the degraded areas are primarily concentrated in the western regions, with fewer in the east. Specifically, the largest conversion is from grassland to unused land, covering 36,169.18 km^2^, followed by the conversion from forestland to grassland (6973.0 km^2^) and from permanent glacier snow to unused land (3640.55 km^2^). Between 2000 and 2020, grassland and permanent glacier snow areas were reduced by 37,016.65 and 4216.21 km^2^, respectively (Table 5). Grassland degradation is mainly concentrated in the upper‐middle region of the study area, while glacier degradation occurs primarily in the western region. Thus, the reduction of grassland and glacier areas, along with the increase in unused land, is the primary causes of the decline in habitat quality in YLZB over the past two decades. This change has led to issues such as desertification, glacier retreat, and grassland degradation in YLZB. Climate warming has caused glaciers to continue retreating, permafrost to melt more rapidly, and soil to be exposed. This results in severe soil erosion and habitat loss, thereby reducing habitat quality (Xu et al. 2017).

### 3.4 The Driving Mechanisms Leading to the Decline in Habitat Quality

#### 3.4.1 Single Factor Analysis

**FIGURE 8:**
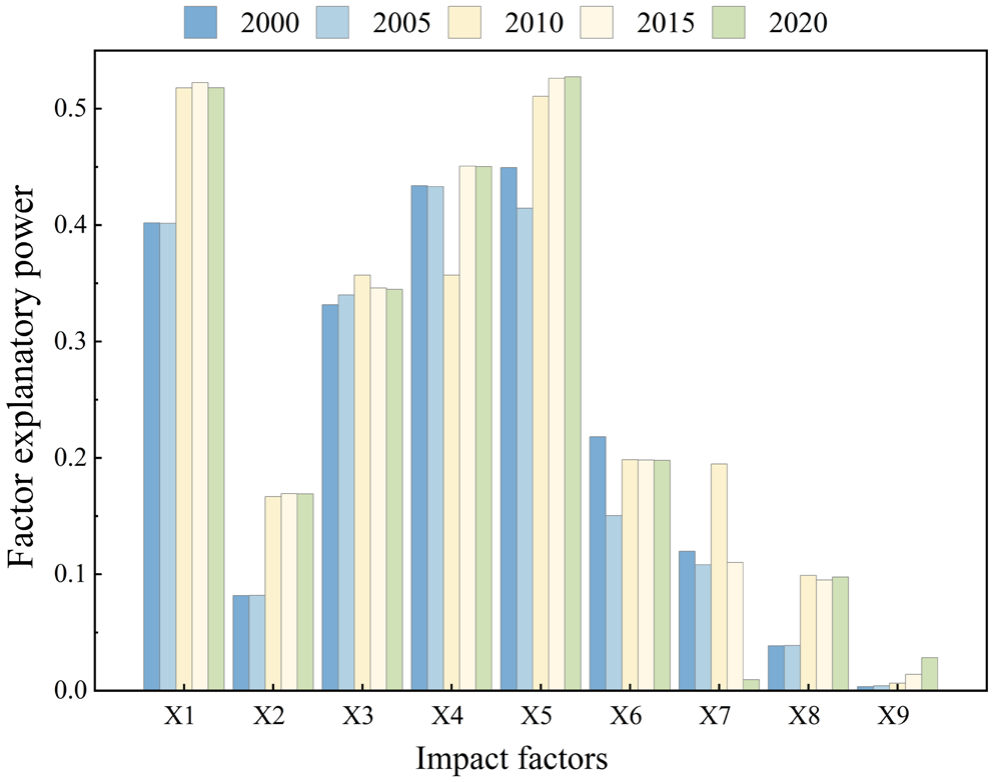
Influence of different factors on habitat quality explained power from 2000 to 2020 (*p* < 0.001). “X1” Indicates elevation, “X2” indicates slope, “X3” indicates annual precipitation, “X4” indicates annual temperature, “X5” indicates NDVI, “X6” indicates soil type, “X7” indicates GDP, “X8” indicates population density, and “X9” indicates nighttime lights.

The contribution rates of various driving factors to the habitat quality of YLZB show strong consistency in 2000, 2005, 2010, 2015, and 2020 (Figure 8). In contrast, factors such as slope, soil type, population density, GDP, and nighttime lights exert comparatively smaller impacts on habitat quality. These influences exhibit significant temporal variability from 2000 to 2020. The intensity of the impact of each factor on habitat quality varies significantly over time. Specifically, elevation (X1) increased from 0.402 in 2000 to 0.518 in 2020. In contrast, precipitation (X3) increased from 2000 to 2010, followed by a decline from 2010 to 2020. Temperature (X4) and NDVI (X5) showed a slight decrease in their q‐values between 2000 and 2005, but they continuously increased from 2005 to 2020.

#### 3.4.2 Interaction Detection

**FIGURE 9:**
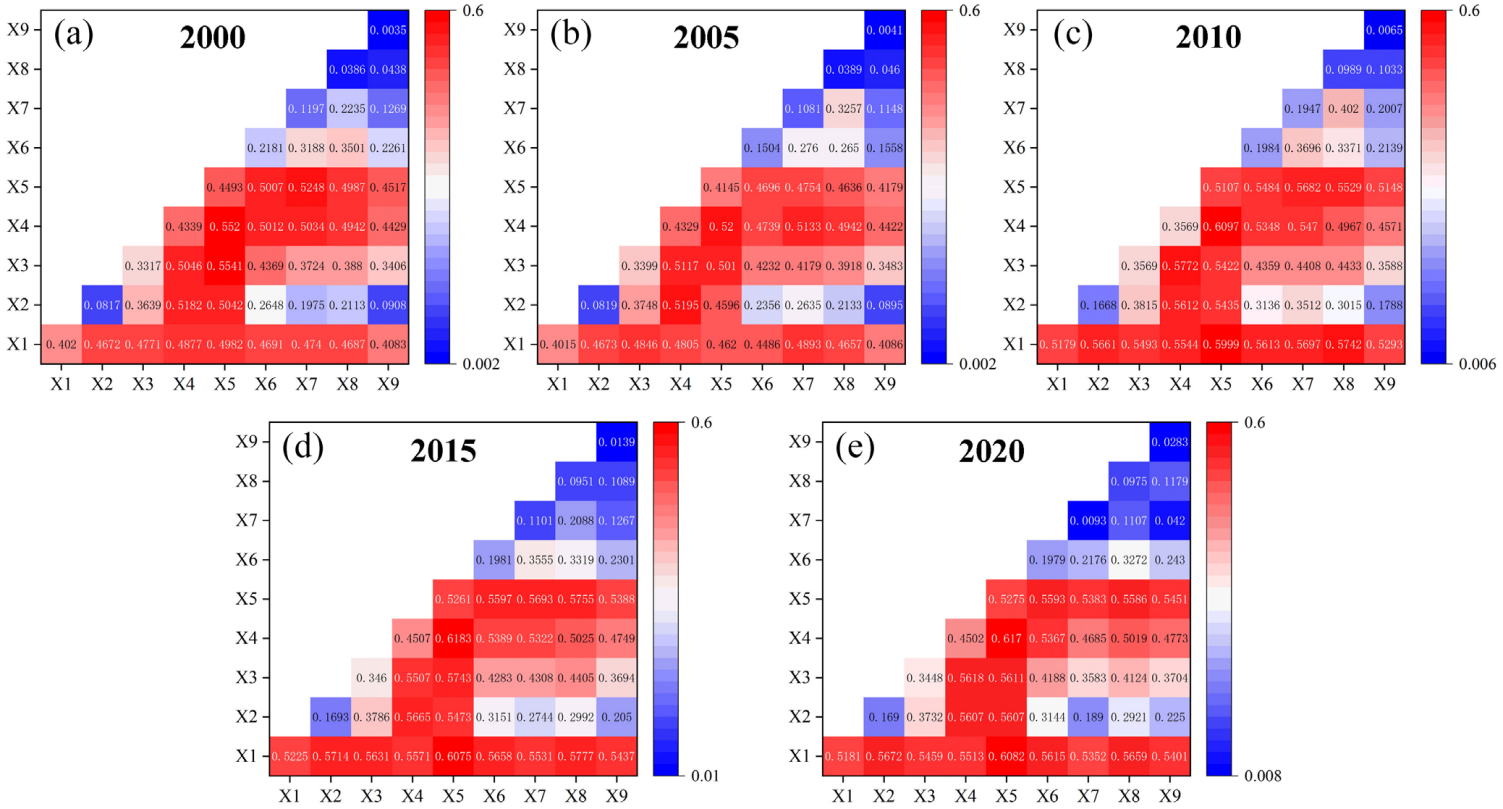
(a–e) Interaction detection results from 2000 to 2020.

Interaction analysis reveals that combinations of different factors synergistically enhance their explanatory power on habitat quality, as illustrated in Figure 9. Throughout the period from 2000 to 2020, interactions involving elevation, NDVI, and other factors demonstrate the highest explanatory rates for spatial variations in habitat quality within the study area. The most influential interaction is observed between NDVI (X5) and annual temperature (X4) (*q* > 0.52), followed by elevation (X1) and annual temperature (X4) (*q* > 0.46). Factors associated with human activities, such as socioeconomic factors, population density, and nighttime lights, significantly amplify their impacts on habitat quality when interacting with natural factors. This underscores that NDVI, elevation, and annual temperature are the primary drivers of spatial differentiation in habitat quality across the study area, with human‐related factors enhancing explanatory power through their interactions with natural variables.

#### 3.4.3 Driving Mechanism Analysis Based on Key Drivers

Based on the above analysis, to further understand the driving mechanism of habitat quality, NDVI, temperature, precipitation, and elevation were selected as key potential variables for subsequent analysis according to the data from the geographic detector. This assessment included metrics such as Average Variance Extracted (AVE), Cronbach's alpha reliability coefficient, and Composite Reliability (CR) (Hair et al. 2019). The internal consistency of variables was evaluated using Composite Reliability (CR), revealing values consistently above 0.7. Additionally, the extracted Average Variance Extracted (AVE) values surpassed 0.5, indicating adequate convergent validity (Nasution et al. 2020). To assess multicollinearity among variables, Variance Inflation Factor (VIF) values were computed, all of which were below 2, confirming negligible covariance among the variables (Thompson et al. 2017). Consequently, all indicators meet established thresholds, affirming the model's soundness and reliability.

**FIGURE 10:**
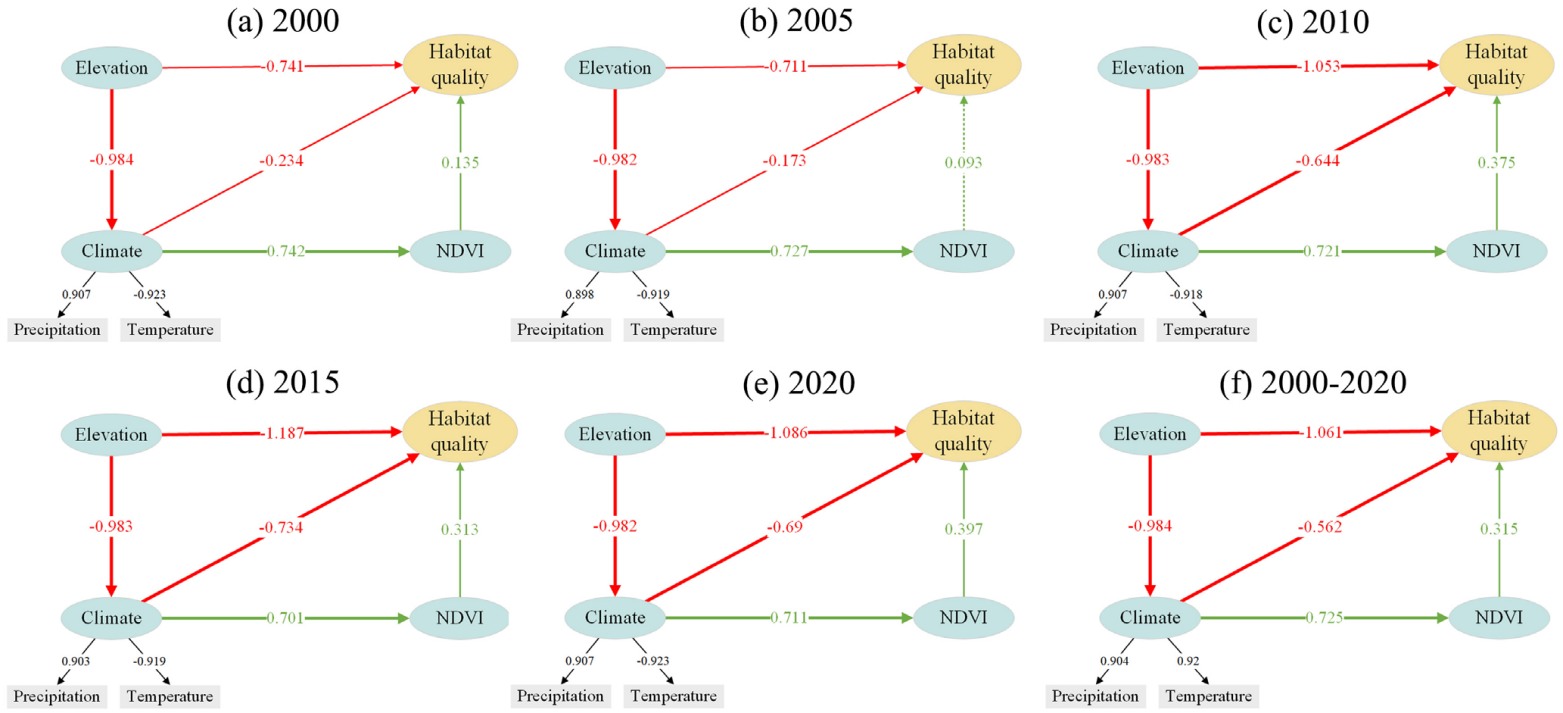
(a–e) PLS‐SEM equation model shows the relationship between climate, altitude, NDVI, and habitat quality from 2000 to 2020, and (f) their average values from 2000 to 2020 (green lines indicate positive correlation, red lines indicate negative correlation, absolute values of path coefficients are represented by thick lines (> 0.4), thin lines (0.1–0.4), and dashed lines (< 0.1)).

Using PLS‐SEM calculations (Figure 10), we obtained the weight coefficients of each observed variable and the path coefficients of the latent variables. The PLS‐SEM framework effectively reflects the impact of various factors on the habitat quality of YLZB. The results indicate a good model fit, with an overall fit between 0.75 and 0.8. During the study period, elevation and climate factors showed a negative correlation with changes in habitat quality. The path coefficients for elevation were −0.741, −0.711, −1.053, −1.187, and −1.086 for the years 2000, 2005, 2010, 2015, and 2020, respectively. For climate, the path coefficients were −0.234, −0.173, −0.644, −0.734, and −0.69. In contrast, NDVI was positively correlated with habitat quality, with path coefficients of 0.135, 0.093, 0.375, 0.313, and 0.397. Additionally, terrain undulation indirectly and negatively impacted habitat quality by influencing the climate, while the climate had a positive indirect impact on habitat quality through its effect on NDVI. From the average values from 2000 to 2020, elevation remained the most influential latent variable, followed by climate and NDVI. Elevation indirectly affected NDVI growth by influencing climate patterns, but the positive impact of NDVI on habitat quality was significantly smaller than the negative impact of elevation and climate on habitat quality.

## 4 Discussion

### 4.1 Effects of Land Use Change on Habitat Quality

The results indicate (Figure 5) that habitat quality in the YLZB shows a gradual improvement from northwest to southeast, which is consistent with previous research findings (Wu et al. 2022). Areas with higher habitat quality are mainly forests, water bodies, and grasslands, while construction land, unused land, and farmland are concentrated in areas with lower habitat quality, which is consistent with the findings of Han, Chen, and Yu (2019). Specifically, the degradation of habitat quality in the western and high‐altitude regions of YLZB is related to several factors: First, the terrain and climatic conditions limit these areas, resulting in significant differences in water and heat conditions. The annual precipitation is sparse, heat is insufficient, and the area belongs to a cold temperate climate, limiting vegetation growth. Secondly, influenced by global warming, surface glaciers are further melting and retreating, and the snow line rises as glaciers shrink. In the western region, most grasslands and permanent glaciers turn into unused land (Table 5), leading to aridification and further affecting the decline in habitat quality (Douville et al. 2021). In addition, policies such as the Western Development Strategy have accelerated urbanization, resulting in the occupation of large amounts of farmland, an increase in construction land, and the migration of agricultural labor from the central and western regions, such as Shigatse, to urban areas (Zhang, Zou, and Zhang 2023; Li et al. 2022). In contrast, the eastern regions, such as the valleys on the southern side of the Nyainqêntanglha Mountains and the source of the Lhasa River, benefit from unique terrain and the influence of the South Asian monsoon, which provides ample water for vegetation growth. Grasslands and forests have natural ecological conditions and rich biodiversity, and areas with high vegetation cover and sufficient precipitation are more suitable for species survival and reproduction, thus having higher habitat quality (Lu, Li, and Gong 2022; Lu, Dai, and Wu 2023). Furthermore, ecological protection projects such as returning farmland to forests and grasslands have promoted vegetation restoration, thereby having a positive impact on habitat quality.

Over the past two decades, the habitat quality index in the YLZB region has declined, but the average habitat quality index remains around 0.56, indicating a moderate level (0.4–0.6), which is similar to the habitat quality indices obtained for other plateau regions by Yu et al. (2023, 2024). Additionally, the habitat quality index in the YLZB region fluctuates significantly, with a major turning point occurring between 2005 and 2010. Around 2010, the habitat quality index experienced a decline followed by a slow recovery. Since 2000, the YLZB region has undergone rapid urbanization and a series of policies (e.g., Western Development Strategy, Belt and Road Initiative, and new urbanization), which promoted industrial structure transformation and population growth, resulting in a shift in land use patterns in the watershed. However, farmland has decreased, and urban and rural construction land has increased significantly, which is consistent with the findings of Zhang, Xu, and Liu (2019). Human activities such as excessive land reclamation, overgrazing, and urbanization have also exacerbated vegetation degradation in the watershed. Particularly after 2010, primary industry activities in the Qinghai‐Tibet region began to decline, while secondary and tertiary industries rapidly increased, promoting a shift in land use from farmland to grassland, forests, and urban areas, while also increasing land use activities (Hao, Zhu, and Cui 2021). Additionally, existing studies have shown that, due to global warming, the average annual temperature in the Tibetan Plateau increases by 0.7°C every 10 years, with the warming trend gradually decreasing from south to north. The decrease in precipitation, influenced by the Tibetan Plateau air mass and the Indian Ocean air currents, leads to a clear warming and drying trend, particularly evident between 2005 and 2010 (Ma et al. 2023; Huang et al. 2019). Therefore, the dramatic changes in land use between 2000 and 2010 directly affected the distribution and quality of habitats. Urbanization, agricultural expansion, overgrazing, and other human activities led to soil erosion, land degradation, and habitat fragmentation, thus impacting habitat quality.

### 4.2 The Effects of Topography and Climate Change on Habitat Quality

**FIGURE 11:**
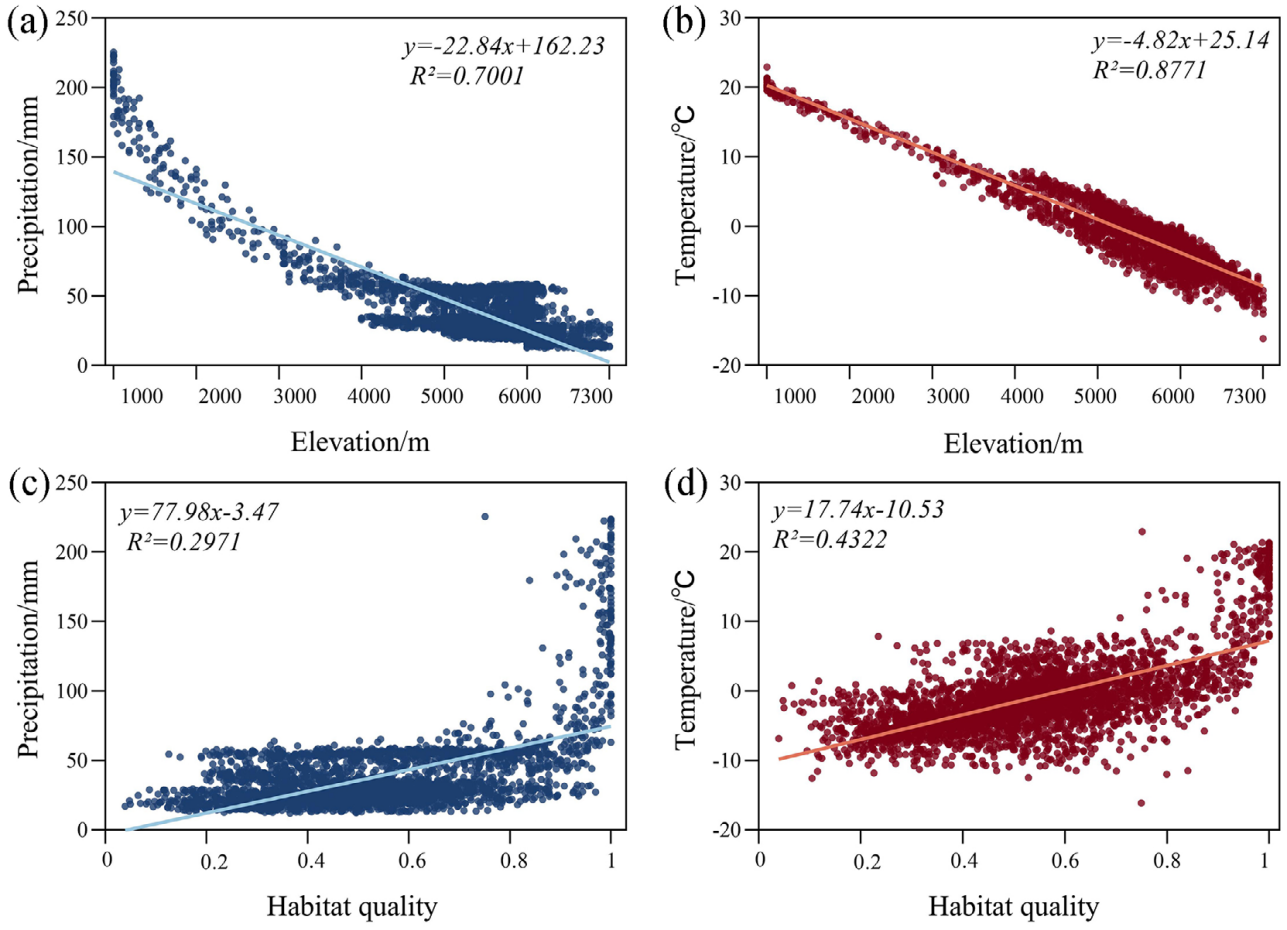
(a) Relationship between precipitation and elevation gradient, and (b) Relationship between temperature and elevation gradient, and (c) Relationship between precipitation and habitat quality, and (d) Relationship between temperature and habitat quality.

PLS‐SEM results indicate that topography has a significant impact on habitat quality changes by influencing climate change and NDVI growth (Figure 10). To further investigate the relationship between climate change, topography, and habitat quality, a univariate linear trend analysis was conducted on the correlation between average annual temperature, precipitation, topography, and habitat quality. To further investigate the relationship between climate change, topography, and habitat quality, a univariate linear trend analysis was conducted on the correlation between average annual temperature, precipitation, topography, and habitat quality. The analysis shows that precipitation, temperature, and elevation have a strong negative correlation, meaning that temperature and humidity decrease as elevation increases (Figure 11). Climate change shows high‐altitude dependence, and the correlation between elevation and temperature is stronger than that with precipitation, which is consistent with the conclusions of You et al. (2021) and Tao et al. (2018). This is due to the diversity of topography, which causes significant climatic variations in the YLZB region. In high‐altitude areas, the air is thin, and the atmosphere's insulating effect on the ground is weak, thus affecting surface temperature and moisture evaporation (Li et al. 2024). In low‐altitude areas, the atmosphere is denser, with relatively higher amounts of water vapor and greenhouse gases such as carbon dioxide. These gases can effectively absorb surface radiation and return it to the ground as atmospheric longwave radiation, providing better insulation. Due to the mountain terrain, warm and humid air is forced to rise. After cooling and condensation at high altitudes, the water vapor forms orographic rainfall, leading to increased precipitation on the southeast windward slopes of the YLZB, while precipitation is relatively lower on the western side, blocked by the mountains, creating arid and semi‐arid climatic zones (Xu et al. 2020; Feng et al. 2020).

### 4.3 Effects of Human Activities on Habitat Quality

The results show (Figures 5 and 6) that habitat quality in the YLZB region declined from 2000 to 2010, and then stabilized and increased after 2010. This change is closely related to human activities such as the management of national parks, ecological protection policies, and ecological restoration projects (Xue 2020). Since the late 1990s, overgrazing in the northwest of the plateau has intensified, with improper grassland utilization leading to a decline in grassland biomass and soil nutrients, causing grassland degradation and weakened ecological restoration capacity, which negatively affects the vegetation growth environment in the YLZB region. During the 2005–2010 period, China was in a phase of rapid development, with the construction of transportation and energy infrastructure, such as the Qinghai‐Tibet Railway, disrupting the surrounding ecological environment. The mining activities led to increased pollutant emissions, and natural disasters such as ice lake outbursts and glacier mudflows frequently occurred, posing serious threats to the YLZB ecosystem (Wang, Qin, and Hu 2023; Cui et al. 2006; Chen et al. 2015).

With the implementation of environmental protection policies and the construction of ecological projects, habitat quality has shown a stable upward trend, with significant results in ecological construction, consistent with the findings reported by Yang et al. (2023). After 2008, the local government restricted slash‐and‐burn farming, reducing smoke from condensation nuclei in the air, closely related to the implementation of regional policies such as vegetation restoration and afforestation. In 2011, Tibet implemented a grazing ban and introduced policies such as the grassland ecological protection subsidy and reward mechanism, along with various grassland ecological protection projects. These measures effectively addressed the issue of overgrazing and improved the recovery capacity of degraded grasslands. In 2012, the Nanshan region successfully piloted artificial afforestation in semi‐arid areas above 3900 m in altitude. In 2013, during Tibet's “12th Five‐Year Plan,” ecological construction policies were implemented, and vegetation gradually recovered (Meng et al. 2021). In recent years, the Tibet Autonomous Region has initiated pilot projects for the construction of national‐level ecological function protection areas, carried out large‐scale natural forest protection in the central basin, strengthened the protection of plateau natural forests, strictly limited logging, and implemented measures such as desertification control, which have curbed the shrinkage of natural forest areas and effectively increased artificial forest areas, thereby increasing vegetation cover and improving habitat quality in certain areas (Yang et al. 2020). By 2018, ecological projects on the Tibetan Plateau covered one‐third of its total area. The vegetation cover in grassland project areas increased by an average of 16.90%, the total carbon storage in natural forest protection areas increased by 27 million tons per year, and the area of desertified land in Tibet decreased by an average of 97.36 km^2^ annually. Therefore, the construction of ecological protection and restoration projects has achieved outstanding results in improving habitat quality in the YLZB region.

The YLZB region, located on the Tibetan Plateau, holds significant ecological protection value. However, with the intensification of climate change, urban expansion, and mineral resource development, the pressure on the habitat quality in the basin will continue to increase. Based on the results of this study and considering the actual situation of the YLZB, the following recommendations are made for the ecological planning and biodiversity conservation of the basin: continue to advance ecological restoration efforts such as desertification, grassland degradation, and black soil wasteland reclamation in the basin, reduce desertified areas, and improve water quality at the source; improve the ecological compensation system, ensuring that compensation funds are directly used for ecological restoration and protection, and establish an effective supervision system; strictly regulate mineral resource development and implement ecological restoration; limit overgrazing and resource extraction, and increase investment in ecological restoration; encourage farmers and herders to participate in ecological restoration projects, improve the regional ecological environment quality, and simultaneously promote income generation for local communities; implement glacier, snow mountain, and permafrost protection, along with real‐time monitoring projects, to address the impact of climate change on the habitat quality of the YLZB.

## 5 Conclusion

This study comprehensively analyzed the spatiotemporal patterns of habitat quality in the YLZB from 2000 to 2020, summarized the findings using spatial distribution maps, and quantified the direct and indirect effects of key driving factors on habitat quality changes using models such as PLS‐SEM, resulting in several important findings:

1. The predominant land use types in the YLZB include forest land, grasslands, and unused lands. Over the two‐decade period, there was a discernible trend of “reduction in two types and increase in five types” of land use, accompanied by significant changes. Specifically, areas of unused land, forest land, water bodies, construction land, and cropland expanded, while grasslands and permanent glaciers and snowfields decreased. Land transitions predominantly involved conversions from grasslands and permanent glaciers and snowfields to unused lands, notably prominent during 2005–2010. These findings advocate for prudent land resource management and accelerated implementation of policies such as the reforestation of farmlands to bolster habitat quality and ensure sustainable development in the YLZB.
2. Habitat quality in the YLZB exhibits a spatial distribution pattern characterized by “high in the southeast, low in the west,” with high‐value areas primarily concentrated in the downstream Nyingchi region and low‐value areas in the upper reaches of high‐altitude regions. Prioritizing ecological restoration and protection efforts in the central and western regions of the basin is recommended. Habitat quality displayed a relatively stable trend from 2000 to 2020, albeit with a slight decline. Approximately 20.48% of the study area experienced persistent degradation in habitat quality, surpassing areas showing improvement. Degradation hotspots are predominantly found in regions like Zhongba and Shigatse in the upper reaches. Given the vulnerability of the plateau ecosystem, measures focusing on vegetation conservation, enhancement of policies such as grazing bans, and restoration of grazing lands to grasslands in alpine regions are crucial to mitigate human disturbances and enhance overall habitat quality in the YLZB.
3. Single‐factor analysis revealed elevation, NDVI (Normalized Difference Vegetation Index), and annual average temperature as primary drivers influencing spatial variations in habitat quality within the YLZB. Conversely, factors like population density, nighttime lights, and GDP exerted minimal impacts on habitat quality. Interaction analysis highlighted synergistic effects among these drivers, with the interaction between annual average temperature and NDVI demonstrating the most pronounced influence. Socioeconomic factors, particularly population density, interact significantly with natural factors, underscoring how environmental conditions establish the ecological foundation for regional habitat quality patterns, while socioeconomic dynamics modulate habitat quality levels through land use changes.
4. The results from PLS‐SEM and correlation analysis show that elevation, climate conditions, and NDVI impact habitat quality with values of −1.06, −0.56, and 0.31, respectively. Temperature and precipitation are dependent on elevation (*R* = −0.937, *R* = −0.837), and elevation indirectly affects vegetation growth through climate change, which in turn worsens habitat quality. Although human activities have exacerbated habitat quality changes to some extent, terrain conditions have dominated the dynamics of habitat quality changes in the YLZB over the past 20 years. The research results provide a basis for decision‐making in watershed land use planning and ecological environmental protection.

## Author Contributions

**Yu Chen:** conceptualization (equal), data curation (equal), formal analysis (lead), investigation (equal), methodology (equal), software (lead), validation (equal), visualization (equal), writing – original draft (lead). **Yujie Kang:** conceptualization (equal), software (equal), visualization (equal). **Jingji Li:** conceptualization (equal), formal analysis (supporting), funding acquisition (equal), investigation (equal), project administration (supporting), supervision (lead), validation (equal), writing – review and editing (equal). **Yanguo Liu:** formal analysis (equal), investigation (equal), methodology (equal), supervision (equal), writing – review and editing (equal). **Qin Liu:** validation (equal), writing – review and editing (equal). **Zhengyu Luo:** conceptualization (equal), investigation (equal), writing – review and editing (equal). **Xiaohui Zhou:** supervision (equal), writing – review and editing (equal). **Tingbin Zhang:** supervision (equal), validation (equal), writing – review and editing (equal). **Guoyan Wang:** writing – review and editing (equal). **Xiaolu Tang:** writing – review and editing (equal). **Xiangjun Pei:** writing – review and editing (equal).

## Conflicts of Interest

The authors declare no conflicts of interest.

## Data Availability

The data that supports the findings of this study and the R code are freely downloadable from Dryad at (https://datadryad.org/stash/share/2R_LHff5xAu7oJV5io4zZmZh81RKfWIHi9pKcA3DBH8).

## References

[ece370807-bib-0001] Aguilar, R. , E. J. Cristobal‐Perez , F. J. Balvino‐Olvera , et al. 2019. “Habitat Fragmentation Reduces Plant Progeny Quality: A Global Synthesis.” Ecology Letters 22, no. 7: 1163–1173. 10.1111/ele.13272. 31087604

[ece370807-bib-0002] Ahmadi Mirghaed, F. , and B. Souri . 2021. “Relationships Between Habitat Quality and Ecological Properties Across Ziarat Basin in Northern Iran.” Environment, Development and Sustainability 23, no. 11: 16192–16207. 10.1007/s10668-021-01343-x.

[ece370807-bib-0003] Bai, L. , C. L. Xiu , X. Feng , et al. 2019. “Influence of Urbanization on Regional Habitat Quality: A Case Study of Chang Chun City Author Links Open Overlay Panel.” Habitat International 93: 102042. 10.1016/j.habitatint.2019.102042.

[ece370807-bib-0004] Berta Aneseyee, A. , T. Noszczyk , T. Soromessa , and E. Elias . 2020. “The InVEST Habitat Quality Model Associated With Land Use/Cover Changes: A Qualitative Case Study of the Winike Watershed in the Omo‐Gibe Basin, Southwest Ethiopia.” Remote Sensing 12, no. 7: 1103. 10.3390/rs12071103.

[ece370807-bib-0005] Chen, B. , H. D. Li , X. Z. Cao , et al. 2016. “Dynamic Changes in Vegetation Coverage in the Yarlung Zangbo River Basin Based on SPOT – VGT NDVI.” [In Chinese.] Mountain Research 34, no. 2: 249–256.

[ece370807-bib-0006] Chen, D. L. , B. Q. Xu , T. D. Yao , et al. 2015. “Assessment of Past, Present and Future Environmental Changes on the Tibetan Plateau.” [In Chinese.] Chinese Science Bulletin 60, no. 32: 3025–3035. +1‐2.

[ece370807-bib-0007] Chen, F. H. , Y. F. Wang , X. L. Zhen , et al. 2021. “Research on Environmental Impacts and Response Strategies on the Tibetan Plateau Under Global Change.” [In Chinese.] China Tibetology 04: 21–28.

[ece370807-bib-0008] Cui, X. F. , H. F. Graf , B. Langmann , et al. 2006. “Climate Impacts of Anthropogenic Land Use Changes on the Tibetan Plateau.” Global and Planetary Change 54, no. 1/2: 33–56. 10.1016/j.gloplacha.2005.07.006.

[ece370807-bib-0009] Dai, L. , S. Li , B. J. Lewis , et al. 2019. “The Influence of Land Use Change on the Spatial–Temporal Variability of Habitat Quality Between 1990 and 2010 in Northeast China.” Journal of Forestry Research 30, no. 6: 2227–2236. 10.1007/s11676-018-0771-x.

[ece370807-bib-0010] Dai, X. A. , J. X. Ma , Y. L. Tang , et al. 2024. “Spatio‐Temporal Dynamics and Attribution Analysis of Vegetation in Gansu Province.” [In Chinese.] Ecology and Environment 33, no. 8: 1163–1173.

[ece370807-bib-0011] Deng, L. C. 2023. Mangrove Ecosystem Health Assessment in Beibu Gulf, Guangxi Based on Multi‐Source Remote Sensing. [In Chinese.]. Gulin University of Technology.

[ece370807-bib-0012] Douville, H. , K. Raghavan , J. Renwick , et al. 2021. “Climate Change 2021: The Physical Science Basis.” In Contribution of Working Group I to the Sixth Assessment Report of the Intergovernmental Panel on Climate Change, 1055–1210. Cambridge, UK: Cambridge University Press. 10.1017/9781009157896.010.

[ece370807-bib-0014] Fan, Y. , and C. Fang . 2022. “Measuring Qinghai‐Tibet Plateau's Sustainability.” Sustainable Cities and Society 85: 104058. 10.1016/j.scs.2022.104058.

[ece370807-bib-0015] Feng, X. L. , H. Y. Shen , W. Z. Li , et al. 2020. “Spatiotemporal Changes for Extreme Precipitation in Wet Season Over the Qinghai‐Tibetan Plateau and the Surroundings During 1961–2017.” [In Chinese.] Plateau Meteorology 39, no. 4: 694–705.

[ece370807-bib-0016] Fu, B. J. 2022. “Ecological and Environmental Effects of Land‐Use Changes in the Loess Plateau of China.” [In Chinese.] Chinese Science Bulletin 67, no. 32: 3769–3779. +3768.

[ece370807-bib-0017] Fu, B. J. , D. Niu , S. D. Zhao , et al. 2005. “Study on Global Change and Terrestrial Ecosystems: History and Prospect.” [In Chinese.] Advances in Earth Science 5: 556–560.

[ece370807-bib-0018] Guo, Z. , L. Zhang , and Y. M. Li . 2010. “Increased Dependence of Humans on Ecosystem Services and Biodiversity.” PLoS One 5, no. 10: e13113. 10.1371/journal.pone.0013113. 20957042 PMC2948508

[ece370807-bib-0019] Hair, J. F. , G. Hult , C. M. Ringle , et al. 2014. “Primer on Partial Least Squares Structural Equation Modeling.” Long Range Planning 46, no. 1–2: 184–185. 10.1016/j.lrp.2013.01.002.

[ece370807-bib-0020] Hair, J. F. , J. J. Risher , M. Sarstedt , and C. M. Ringle . 2019. “When to Use and How to Report the Results of PLS‐SEM.” European Business Review 31, no. 1: 2–24. 10.1108/EBR-11-2018-0203.

[ece370807-bib-0021] Han, Y. , K. Chen , and D. Yu . 2019. “Evaluation on the Impact of Land Use Change on Habitat Quality in Qinghai Lake Basin.” [In Chinese.] Ecology and Environmental Sciences 28: 2035–2044.

[ece370807-bib-0022] Hao, S. , F. Zhu , and Y. Cui . 2021. “Land Use and Land Cover Change Detection and Spatial Distribution on the Tibetan Plateau.” Scientific Reports 11: 7531. 10.1038/s41598-021-87215-w. 33824387 PMC8024275

[ece370807-bib-0023] Hou, Y. , W. Zhao , Y. Liu , et al. 2021. “Relationships of Multiple Landscape Services and Their Influencing Factors on the Qinghai–Tibet Plateau.” Landscape Ecology 36: 1987–2005. 10.1007/s10980-020-01140-3.

[ece370807-bib-0024] Huang, J. , F. Zheng , X. Dong , and X. C. Wang . 2023. “Exploring the Complex Trade‐Offs and Synergies Among Ecosystem Services in the Tibet Autonomous Region.” Journal of Cleaner Production 384: 135483. 10.1016/j.jclepro.2022.135483.

[ece370807-bib-0025] Huang, X. H. , Y. Z. Zhou , J. P. Fang , and L. Hou . 2019. “Climate Change has More Adverse Impacts on the Higher Mountain Communities Than the Lower Ones: People's Perception From the Northern Himalayas.” Journal of Mountain Science 16, no. 11: 2625–2639. 10.1007/s11629-018-5352-0.

[ece370807-bib-0026] Jia, T. C. , X. W. Hu , H. J. Yang , et al. 2024. “Spatial Response of Habitat Quality to Climate Change and Human Activities: Taking the Qinghai—Tibet Plateau as an Example.” [In Chinese.] Journal of Soil and Water Conservation 38, no. 5.

[ece370807-bib-0028] Li, H. Q. , Y. Yang , and J. H. Zhang . 2023. “Construction of Ecological Security Pattern in ShannanWide Valley Basin of Yarlung Zangbo River.” [In Chinese.] Arid Land Geography 46, no. 9: 1503–1513.

[ece370807-bib-0029] Li, M. Y. , Y. Zhou , P. N. Xiao , et al. 2021. “Evolution of Habitat Quality and Its Topographic Gradient Effect in Northwest Hubei Province From 2000 to 2020 Based on the Invest Model.” Land 10, no. 8: 857. 10.3390/land10080857.

[ece370807-bib-0030] Li, P. , D. Zuo , Z. Xu , et al. 2021. “Dynamic Changes of Land Use/Cover and Landscape Pattern in a Typical Alpine River Basin of the Qinghai‐Tibet Plateau, China.” Land Degradation & Development 32, no. 15: 4327–4339. 10.1002/ldr.4039.

[ece370807-bib-0031] Li, X. , Y. Chen , L. Xu , P. Li , and R. Zhang . 2022. “Transformation of Farmland Use and Driving Mechanism in Xinjiang Since China's Western Development Policy.” Frontiers in Ecology and Evolution 10: 942065. 10.3389/fevo.2022.942065.

[ece370807-bib-0032] Li, X. Y. , G. C. Cao , Z. Y. Chen , et al. 2024. “Spatial‐Temporal Characteristics and Influencing Factors of Land Surface Temperature on the Southern Slope of Qilian Mountains From 2001 to 2022.” [In Chinese.] Journal of Desert Research 44, no. 5: 84–94.

[ece370807-bib-0034] Liu, X. T. , W. C. He , H. Peng , et al. 2023. “Rare Earth Elements in the Upper Reaches of Yarlung Zangbo River.” [In Chinese.] China Environmental Science 43, no. 6: 3068–3076.

[ece370807-bib-0035] Liu, X. W. , Z. X. Xu , and D. Z. Peng . 2019. “Spatio‐Temporal Patterns of Vegetation in the Yarlung Zangbo River, China During 1998–2014.” Sustainability 11, no. 16: 4334. 10.3390/su11164334.

[ece370807-bib-0036] Lu, R. , E. Dai , and C. Wu . 2023. “Spatial and Temporal Evolution Characteristics and Driving Factors of Soil Conservation Services on the Qinghai‐Tibet Plateau.” Catena 221: 106766. 10.1016/j.catena.2022.106766.

[ece370807-bib-0037] Lu, Y. , T. Li , and J. Gong . 2022. “Attribution of Habitat Quality in Different Geomorphological Types in Guangdong Province.” [In Chinese.] Ecological Sciences 42, no. 17: 6997–7010.

[ece370807-bib-0038] Ma, F. L. , X. W. Liu , Y. Duo , et al. 2023. “Effects of Daily Variation of Hydro‐Thermal Factors on Alpine Grassland Productivity on the Qinghai‐Tibet Plateau.” [In Chinese.] Acta Ecologica Sinica 43, no. 9: 3719–3728.

[ece370807-bib-0039] Meng, Q. B. , Y. L. Liu , Q. Ju , et al. 2021. “Vegetation Change and Its Response to Climate Change in the Yarlung Zangbo River Basin in the Past 18 Years.” [In Chinese.] South‐To‐North Water Transfers and Water Science & Technology 19, no. 3: 539–550.

[ece370807-bib-0040] Mengist, W. , T. Soromessa , and G. L. Feyisa . 2021. “Landscape Change Effects on Habitat Quality in a Forest Biosphere Reserve: Implications for the Conservation of Native Habitats.” Journal of Cleaner Production 329: 129778. 10.1016/j.jclepro.2021.129778.

[ece370807-bib-0041] Nasution, M. I. , M. Fahmi , M. Jufrizen , et al. 2020. “The Quality of Small and Medium Enterprises Performance Using the Structural Equation Model‐Part Least Square (SEM‐PLS).” Journal of Physics Conference Series 1477, no. 5: 52052. 10.1088/1742-6596/1477/5/052052.

[ece370807-bib-0042] Nematollahi, S. , S. Fakheran , F. Kienast , and A. Jafari . 2020. “Application of InVEST Habitat Quality Module in Spatially Vulnerability Assessment of Natural Habitats (Case Study: Chaharmahal and Bakhtiari Province, Iran).” Environmental Monitoring and Assessment 192, no. 8: 487. 10.1007/s10661-020-08460-6. 32621254

[ece370807-bib-0043] Pritchard, H. D. 2019. “Asia's Shrinking Glaciers Protect Large Populations From Drought Stress.” Nature 569: 649–654. 10.1038/s41586-019-1240-1. 31142854

[ece370807-bib-0044] Richter, N. F. , G. Cepeda , J. L. Rold'an , et al. 2015. “European Management Research Using Partial Least Squares Structural Equation Modeling (PLS‐SEM).” European Management Journal 33, no. 1: 1–3. 10.1016/j.emj.2016.08.001.

[ece370807-bib-0045] Shang, J. , H. S. Cai , Y. Long , et al. 2021. “Temporal‐Spatial Distribution and Transition of Habitat Quality in Poyang Lake Region Based on InVEST Model.” [In Chinese.] Resources and Environment in the Yangtze Basin 30, no. 8: 1901–1915.

[ece370807-bib-0046] Sharp, R. , H. T. Tallis , T. Ricketts , et al. 2018. InVEST 3.2. 0 User's Guide. The Natural Capital Project. Stanford University. 10.13140/RG.2.2.32693.78567.

[ece370807-bib-0047] Shi, D. , T. Lee , and A. Maydeu‐Olivares . 2019. “Understanding the Model Size Effect on SEM Fit Indices.” Educational and Psychological Measurement 79, no. 2: 310–334. 10.1177/0013164418783530. 30911195 PMC6425088

[ece370807-bib-0048] Song, Y. , M. Wang , X. Sun , and Z. Fan . 2021. “Quantitative Assessment of the Habitat Quality Dynamics in Yellow River Basin, China.” Environmental Monitoring and Assessment 193: 614. 10.1007/s10661-021-09404-4. 34468858

[ece370807-bib-0049] Song, Y. Z. , J. F. Wang , Y. Ge , et al. 2020. “An Optimal Parameters‐Based Geographical Detector Model Enhances Geographic Characteristics of Explanatory Variables for Spatial Heterogeneity Analysis: Cases With Different Types of Spatial Data.” GIScience & Remote Sensing 57: 593–610. 10.1080/15481603.2020.1760434.

[ece370807-bib-0050] Tao, J. , T. Q. Xu , J. W. Dong , et al. 2018. “Elevation‐Dependent Effects of Climate Change on Vegetation Greenness in the High Mountains of Southwest China During 1982–2013.” International Journal of Climatology 38: 2029–2038. 10.1002/joc.5314.

[ece370807-bib-0051] Thompson, C. G. , R. S. Kim , A. M. Aloe , and B. J. Becker . 2017. “Extracting the Variance Inflation Factor and Other Multicollinearity Diagnostics From Typical Regression Results.” Basic and Applied Social Psychology 39: 81–90. 10.1080/01973533.2016.1277529.

[ece370807-bib-0052] Wang, J. F. , and C. D. Xu . 2017. “Geodetector: Principle and Prospective.” [In Chinese.] Acta Geographica Sinica 72, no. 1: 116–134.

[ece370807-bib-0053] Wang, P. , S. Qin , and h. Hu . 2023. “Spatial‐Temporal Evolution Characteristics of Land Use Change and Habitat Quality in the Lhasa River Basin Over the Past Three Decades.” [In Chinese.] Arid Zone Research 40, no. 3: 492–503.

[ece370807-bib-0054] Wang, Y. , X. F. Wang , L. C. Yin , et al. 2021. “Determination of Conservation Priority Areas in Qinghai Tibet Plateau Based on Ecosystem Services.” Environmental Science & Policy 124: 553–566. 10.1016/j.envsci.2021.07.019.

[ece370807-bib-0055] Wei, Y. , X. Zhu , Y. Li , T. Yao , and Y. Tao . 2019. “Influential Factors of National and Regional CO2 Emission in China Based on Combined Model of DPSIR and PLS‐SEM.” Journal of Cleaner Production 212: 698–712. 10.1016/j.jclepro.2018.11.155.

[ece370807-bib-0056] Wu, J. , G. Wang , W. Chen , S. Pan , and J. Zeng . 2022. “Terrain Gradient Variations in the Ecosystem Services Value of the Qinghai‐Tibet Plateau, China.” Global Ecology and Conservation 34: e02008. 10.1016/j.gecco.2022.e02008.

[ece370807-bib-0057] Xiang, Q. , A. Kan , X. Yu , et al. 2023. “Assessment of Topographic Effect on Habitat Quality in Mountainous Area Using InVEST Model.” Land 12, no. 1: 186. 10.3390/land12010186.

[ece370807-bib-0058] Xu, J. W. , Y. H. Gao , B. F. Peng , et al. 2020. “Change Characteristics of Precipitation and Its Cause During 1979–2016 Over the Qinghai‐Tibetan Plateau.” [In Chinese.] Plateau Meteorology 39, no. 2: 234–244.

[ece370807-bib-0059] Xu, R. , H. C. Hu , F. Tian , et al. 2019. “Projected Climate Change Impacts on Future Streamflow of the Yarlung Tsangpo‐Brahmaputra River.” Global and Planetary Change 175: 144–159. 10.1016/j.gloplacha.2019.01.012.

[ece370807-bib-0060] Xu, S. , Y. Y. Zhang , M. Dou , et al. 2017. “Spatial Distribution of Land Use Change in the Yangtze River Basin and the Impact on Runoff.” Progress in Geography 36, no. 4: 426–436. 10.18306/dlkxjz.2017.04.004.

[ece370807-bib-0081] Xu, J. W. , Y. H. Gao , B. F. Peng , et al., 2020. “Change Characteristics of Precipitation and its Cause During 1979‐2016 over the Qinghai‐Tibetan Plateau.” Plateau Meteorology 39, no. 2: 234–244.

[ece370807-bib-0061] Xu, Z. X. , C. G. Ban , and R. Zhang . 2022. “Evolution Laws and Attribution Analysis in the Yarlung Zangbo River.” [In Chinese.] Advances in Water Science 33, no. 4: 519–530.

[ece370807-bib-0062] Xue, R. Z. 2020. “Analysis on Spatial and Temporal Changes of Regional Habitat Quality Based on the Spatial Pattern Reconstruction of Land Use.” [In Chinese.] Acta Geographica Sinica 75, no. 1.

[ece370807-bib-0064] Yang, P. , X. H. Wei , Y. X. Dong , et al. 2020. “Progress on Sandy Desertification Research and Future Combating Idea in Tibet, China.” [In Chinese.] Bulletin of the Chinese Academy of Sciences 35, no. 6: 699–708.

[ece370807-bib-0065] Yang, W. P. , H. Y. Shao , J. R. Huang , et al. 2023. “Habitat Quality Assesment of Qinghai‐Tibet Plateau Natonal Park Cluster.” [In Chinese.] National Park 1, no. 2: 99–109.

[ece370807-bib-0066] Yang, Z. P. , J. W. Xu , X. H. Feng , et al. 2018. “Effects of Land Use Change on Habitat Based on InVEST Model in Northeast China.” [In Chinese.] Ecologic Science 37, no. 6: 139–147.

[ece370807-bib-0067] You, Q. L. , S. C. Kang , J. D. Li , et al. 2021. “Several Research Frontiers of Climate Change Over the Tibetan Plateau.” [In Chinese.] Journal of Glaciology and Geocryology 43, no. 3: 885–901.

[ece370807-bib-0068] Yu, H. , X. Qian , H. Jing , and Y. Liu . 2024. “Spatiotemporal Evolution Characteristics and the Driving Force of Habitat Quality in the Qinghai–Tibet Plateau in Topographic View (2000–2020).” Frontiers in Ecology and Evolution 12: 1345665. 10.3389/fevo.2024.1345665.

[ece370807-bib-0069] Yu, N. , Y. Z. Xiao , M. Y. Yan , et al. 2023. “Assessing the Impacts of Historical and Future Land‐Use/Cover Change on Habitat Quality in the Urbanizing Lhasa River Basin on the Tibetan Plateau.” Ecological Indicators 148: 110147. 10.1016/j.ecolind.2023.110147.

[ece370807-bib-0070] Yu, S. H. , and X. J. Zhang . 2020. “Analysis on the Characteristics and Trend of Temperature Change in the Yarlung Zangbo River.” [In Chinese.] Journal of Agricultural Catastrophology 10, no. 4: 50–51.

[ece370807-bib-0071] Yu, W. , R. Ji , X. Han , et al. 2020. “Evaluation of the Biodiversity Conservation Function in Liaohe Delta Wetland, Northeastern China.” Journal of Meteorological Research 34, no. 4: 798–805. 10.1007/s13351-020-9186-7.

[ece370807-bib-0072] Zhang, H. J. , Y. Gao , Y. W. Hua , et al. 2019. “Response of a SolVES Model Value Transfer Method to Different Spatial Scales.” Acta Ecologica Sinica 39, no. 24: 9233–9245. (in Chinese).

[ece370807-bib-0073] Zhang, J. , J. L. Zou , and K. Zhang . 2023. “A Comprehensive Model of Basin Ecological Compensation Funds—A Case Study of the Yellow River Basin in China.” Frontiers in Environmental Science 11: 1119576. 10.3389/fenvs.2023.1119576.

[ece370807-bib-0074] Zhang, R. , Z. X. Xu , and X. W. Liu . 2019. “Spatiotemporal Characteristics of Land Use /Cover Change for the From 1980 to 2015.” [In Chinese.] China Rural Water and Hydropower 3: 106–111.

[ece370807-bib-0075] Zhang, T. Y. , S. Shen , C. X. Cheng , et al. 2023. “Dynamic Changes and Influencing Factors of Farmland Pattern in Yarlung Zangbo River Basin From 2000 to 2015.” [In Chinese.] Journal of Beijing Normal University 59, no. 1: 136–146.

[ece370807-bib-0076] Zhang, Y. L. , L. S. Liu , Z. F. Wang , et al. 2019. “Spatial and Temporal Characteristics of Land Use and Cover Changes in the Tibetan Plateau.” [In Chinese.] Chinese Science Bulletin 64: 2865–2875.

[ece370807-bib-0077] Zheng, J. C. , B. G. Xie , and X. B. You . 2022. “Evolution of Habitat Quality and Its Influencing Factors in the Different Terrain Gradient of the Guangdong‐Hong Kong‐Macao Greater Bay Area From 1980 to 2020.” [In Chinese.] Economic Geography 42, no. 8: 41–50.

[ece370807-bib-0078] Zheng, J. C. , B. G. Xie , X. B. You , et al. 2022. “Spatio‐Temporal Characteristics of Habitat Quality Based on Land‐Use Changes in Guangdong Province.” [In Chinese.] Acta Ecologica Sinica 42, no. 17: 6997–7010.

[ece370807-bib-0079] Zheng, L. , Y. Wang , and J. Li . 2023. “Quantifying the Spatial Impact of Landscape Fragmentation on Habitat Quality: A Multi‐Temporal Dimensional Comparison Between the Yangtze River Economic Belt and Yellow River Basin of China.” Land Use Policy 125: 106463. 10.1016/j.landusepol.2022.106463.

[ece370807-bib-0080] Zheng, Y. P. , J. H. Zhang , H. W. Tian , et al. 2024. “Spatio‐Temporal Characteristics of Habitat Quality and Natural‐Human Driven Mechanism in Dabie Mountain Area.” [In Chinese.] Environmental Sciences 45, no. 4: 2268–2279.

